# Imaging as a guide to tissue sampling

**DOI:** 10.1186/1470-7330-14-S1-O7

**Published:** 2014-10-09

**Authors:** Leslie E Quint

**Affiliations:** 1 Department of Radiology, University of Michigan Health System, Ann Arbor, MI 48109-5030 USA

## 

When assessing a patient with lung cancer, it is important to stage the tumor in order to determine prognosis and direct appropriate therapy. Although imaging findings, particularly with CT and PET, can suggest the correct tumor stage, imaging is imperfect in this regard; enlarged and/or hypermetabolic lymph nodes may require sampling for confirmation of presumed tumor stage. A major role for imaging is to direct the most optimal method of tissue sampling in order to establish the highest possible tumor stage, so that proper therapy may be instituted [1]. Lymph node biopsies may be performed using mediastinoscopy for lymph nodes that are adjacent to the trachea or carina; bronchoscopy with endobronchial ultrasound for lymph nodes adjacent to the trachea, carina, mainstem bronchi and more peripheral airways; video assisted thoracoscopic surgery (VATS) for lesions adjacent to the pleural surfaces; Chamberlain procedure for lymph nodes in the aortopulmonary window and anterior paraaortic regions; endoscopic ultrasound (EUS) for nodes adjacent to the esophagus; ultrasonography for nodes in the neck and supraclavicular regions; and CT biopsy for large, accessible nodes. Selection of the best method for obtaining a tissue sample necessitates consideration of various factors, including the location of the lesion, the need for sampling of single vs. multiple lymph node stations, the amount of tissue that is necessary to make a confident diagnosis, the expected diagnostic yield and accuracy of the technique, the cost and availability of the procedure at the patient’s institution, the expertise of the physicians, and the safety and risks involved.
